# Regulatory Mechanisms and Clinical Applications of the Long Non-coding RNA PVT1 in Cancer Treatment

**DOI:** 10.3389/fonc.2019.00787

**Published:** 2019-08-21

**Authors:** Meng-Yuan Li, Xiao-Huan Tang, Yan Fu, Tie-Jun Wang, Jia-Ming Zhu

**Affiliations:** ^1^Department of Radiotherapy, The Second Hospital of Jilin University, Changchun, China; ^2^Department of Gastrointestinal Nutrition and Hernia Surgery, The Second Hospital of Jilin University, Changchun, China; ^3^Department of General Surgery, Huashan Hospital, Fudan University, Shanghai, China

**Keywords:** cancer, lncRNA, PVT1, regulatory mechanism, therapeutic target

## Abstract

Cancer is the second leading cause of death worldwide, and no obvious decline in incidence and mortality has occurred in recent years. It is imperative to further investigate the mechanisms underlying tumor progression. Long non-coding RNAs have received considerable attention in recent years because of their major regulatory roles in gene expression. Among them, PVT1 is well-studied, and substantial evidence indicates that PVT1 plays critical roles in the onset and development of cancers. Normally, PVT1 acts as an oncogenic factor by promoting cancer cell proliferation, invasion, metastasis, and drug resistance. Herein, we summarize current knowledge regarding the regulatory effects of PVT1 in cancer progression, as well as the related underlying mechanisms, such as interaction with Myc, modulation of miRNAs, and regulation of gene transcription and protein expression. In extracellular fluid, PVT1 mainly promotes cancer initiation, and it normally enhances cellular cancer characteristics in the cytoplasm and cell nucleus. Regarding clinical applications, its role in drug resistance and its potential use as a diagnostic and prognostic marker have received increasing attention. We hope that this review will contribute to a better understanding of the regulatory role of PVT1 in cancer progression, paving the way for the development of PVT1-based therapeutic approaches in cancer treatment.

## Introduction

Over the past several decades, cancer has remained a major public health challenge worldwide despite tremendous advances in therapies, such as surgery, chemotherapy, radiotherapy and immunotherapy (1, 2). The poor prognosis is mainly due to frequent relapse, metastasis and drug resistance, and the low rate of early stage diagnosis is closely associated with these aspects. There remains a need to explore mechanisms of cancer initiation and progression and to develop more effective strategies for diagnosis and treatment to improve the prognosis and quality of life of cancer patients (3).

Previous genome research studies have focused mainly on protein-coding genes, whereas non-protein-encoding genes were regarded as junk DNA. Over the past decade, the development of high-throughput technologies has fostered an in-depth examination of the non-encoding genome. Less than 2% of the mammalian genome has been found to possess protein-coding ability, and non-coding RNAs (ncRNAs) may account for most of the mammalian genome (4). These findings have altered the perception of ncRNAs being a product of “junk” transcription, and ncRNAs are now considered gene expression regulators. Long non-coding RNA (lncRNAs) are ncRNA transcripts of more than 200 nucleotides. Accumulating evidence indicates the regulatory roles of ncRNA in cellular processes and pathways in developmental and pathological contexts. LncRNAs cannot be translated into proteins but have been proposed to be important regulators of gene expression through transcriptional regulation in cis or trans, regulation of protein or RNA molecules, and organization of nuclear domains (5). Emerging studies indicate the dysregulation of lncRNA in cancer, and these lncRNAs are closely associated with cancer progression (6).

Among all dysregulated lncRNAs, PVT1 is well studied and is regarded as an important oncogenic factor in cancer (3). The *PVT1* gene, located at locus 8q24, has a close functional relationship with *myelocytomatosis* (*Myc*), which exerts oncogenic effects in various cancers (7). PVT1 contributes to the overexpression of Myc, thereby inducing the onset and progression of cancer (8). Recently, several studies have suggested that PVT1 and Myc may exert synergistic effects in cancer initiation. Additionally, the overexpression and regulatory effects of PVT1 in human tumors, such as cervical cancer (9), prostate cancer (10), melanoma (11), and gastric cancer (12), have been described. Moreover, genome-wide screening has identified PVT1 as a regulator of chemotherapy resistance, and PVT1 also plays a vital role in cancer drug resistance (13). On the basis of these mechanisms, PVT1 appears to have a high potential for clinical application in cancer treatment. A positive correlation between PVT1 and patient prognosis has been observed, thus indicating the potential of PVT1 to serve as a prognostic indicator for cancer patients (14). In colorectal cancer, patients with elevated expression of PVT1 also show poorer prognosis (15). Yang et al. have reported that serum PVT1 can serve as a biomarker for diagnosis of cervical cancer (16). Together, these results suggest that PVT1 may have a critical role in cancer progression, and might serve as a diagnostic or prognostic indicator for cancer.

In this review, we summarize current understanding of the underlying mechanisms and clinical applications of the stimulatory effect of PVT1 in various cancers, to support a better understanding of the regulatory role of PVT1 and to facilitate the application of PVT1-targeting therapies as an anti-cancer strategy.

## Mechanisms

**Figure 1:**
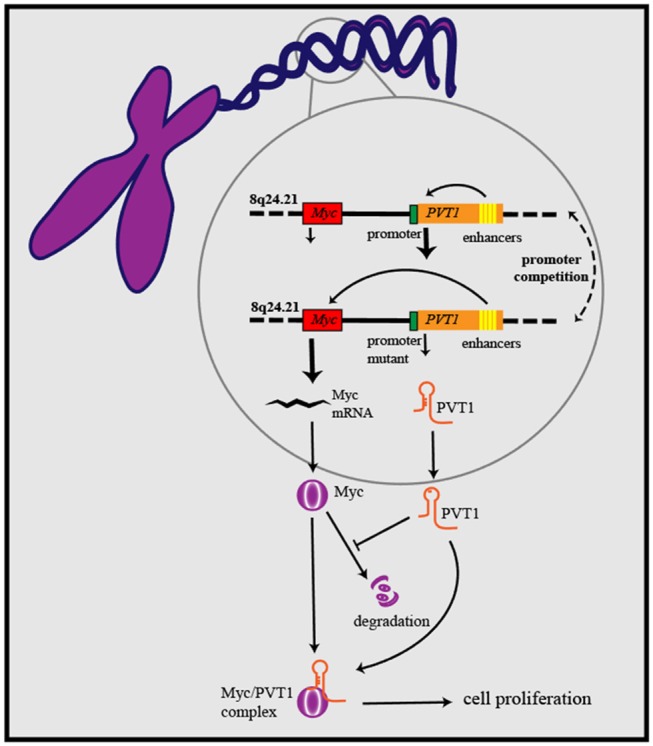
PVT1/Myc co-operation exerts oncogenic effects: PVT1 promoter inhibits Myc transcription from the same chromosome through promoter competition, further to regulate Myc expression and influence cancer development. PVT1 upregulates the level of Myc protein and enhances its stability by inhibiting its degradation. On the basis of forming a complex with Myc, PVT1 promotes cancer cell proliferation.

**Figure 2:**
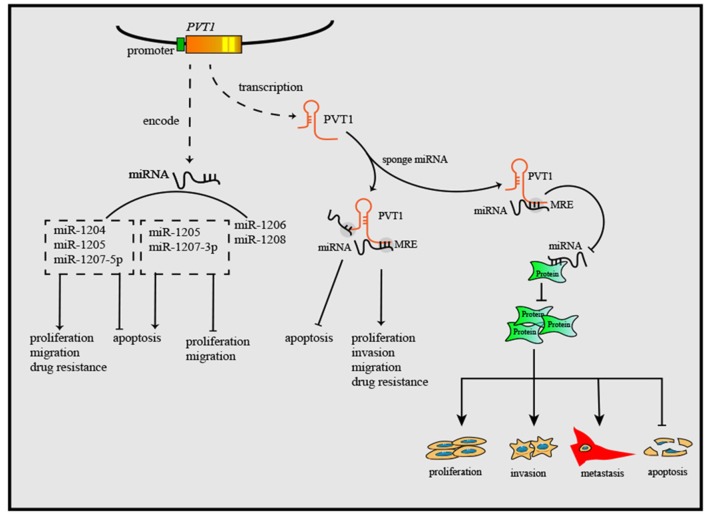
PVT1 gene exerts anti-tumor or oncogenic effects by encoding six miRNAs. Through sponging miRNAs, PVT1 regulates cancer cell proliferation, apoptosis, invasion, and migration. Additionally, PVT1 also can modulate the onset and progression of cancers by regulating miRNA-related proteins, after sponging corresponding miRNAs.

**Figure 3:**
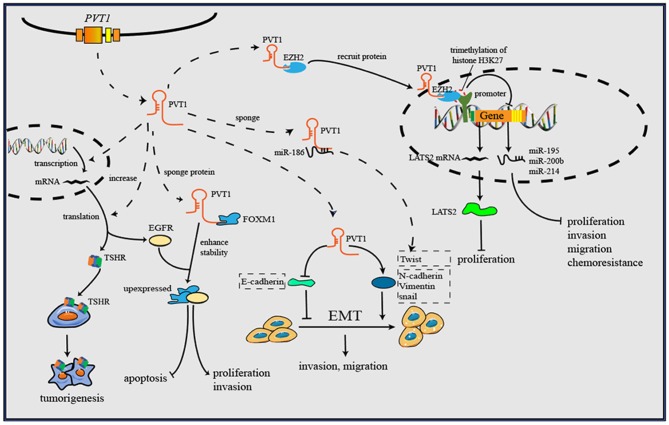
PVT1 forms a complex with EZH2 and then recruits EZH2 to the promoter regions of some anti-tumor genes, then increases the trimethylation of histone H3K27, and downregulates the levels of gene transcription. Through downregulating E-cadherin but upregulating vimentin, snail and Twist1, PVT1 induces EMT, thus promoting cancer invasion and metastasis. By binding to corresponding proteins, PVT1 can increase their expression or enhance their stability, and further to exert oncogenic effects. Additionally, PVT1 promotes tumorigenesis by upregulating the expression level of TSHR in in thyroid cancer.

As research has progressed, mechanisms utilized by PVT1 to exert regulatory effects on cancers progression have increasingly been identified (Table 1). First, the *Myc* gene received substantial attention because of its co-localization with the *PVT1* gene in the 8q24.21 region, and studies have demonstrated the oncogenic role of Myc/PVT1 co-operation (Figure 1). In addition, PVT1 interacts with miRNAs (Figure 2) and regulates gene transcription and protein expression (Figure 3), thereby exerting effects on cancer cells. Furthermore, there are six associated miRNA sequences in the *PVT1* region, which exert regulatory effects. We summarize these mechanisms below.

**Table 1:** Recent studies on the lncRNA PVT1 in cancers.

**First author**	**Year**	**Cancer**	**PVT1-binded miRNAs**	**Regulated proteins**	**Functional role**	**References**
Chingju Shen	2017	Cervical cancer	miR-195	EMT, EZH2	Chemosensitivity↓	(9)
Hongtao Liu	2016	Prostate cancer	miR-146a	–	Pro↑apo↓	(10)
Xiangjun Chen	2017	Melanoma	–	–	Pro↑mig↑	(11)
Ting Li	2017	Gastric cancer	microRNA-152	CD151, FGF2	–	(12)
Chengsuo Huang	2016	Small cell lung cancer	–	–	Inv↑mig↑	(14)
Y Takahashi	2014	Colorectal cancer	–	TGF-β	Apo↓	(15)
J.-P. Yang	2016	Cervical cancer	–	–	–	(16)
Aaron L. Sarver	2016	Breast cancer	–	RSPO1	–	(17)
Chen Yan	2017	Breast cancer	miR-1207-5p	STAT6	Pro↑	(18)
Dapeng Wu	2017	Non-small cell lung cancer	miR-195	-	Radiosensitivity↓	(19)
Quan Zhou	2016	Osteosarcoma	miR-195	BCL-2, CCND1	Pro↑apo↓inv↑mig↑	(20)
				FASN		
Yali Gao	2017	Cervical cancer	miR-424	–	Pro↑inv↑mig↑	(21)
Yanjie Han	2019	Glioma	miR-424	–	Pro↑inv↑mig↑	(22)
				–		
Dong Guo	2018	Non-small-cell lung cancer	miR-497	–	Pro↑inv↑apo↓	(23)
Rui Zhang	2018	Colon cancer	miR-26b	–	Pro↑inv↑mig↓	(24)
Baojuan Wang	2018	Melanoma	miR-26b	–	Pro↑apo↓	(25)
Qi Yang	2018	Ovarian cancer	miR-133a	–	Pro↑inv↑	(26)
Chunhong Wang	2018	Non-small cell lung cancer	miR-199a-5p	HIF-1α	Pro↑	(27)
Tao Huang	2017	Gastric cancer	miR-186	HIF-2α	Pro↑inv↑	(28)
X Yu	2018	Colon cancer	miR-30d-5p	RUNX2	Pro↑mig↑	(29)
Jie Chai	2018	Colorectal cancer	miR-455	RUNX2	Pro↑inv↑mig↑	(30)
Wei Chen	2017	Non-small cell lung cancer	miR-200a	MMP9	Inv↑	(31)
			miR-200b			
Liang Zhao	2018	Pancreatic cancer	miR-448	SERBP1	Inv↑mig↑	(32)
Z Tian	2019	Bladder cancer	miR-31	CDK1	Pro↑inv↑mig↑	(33)
Jingyi Song	2017	Osteosarcoma	miR-497	HK2	Pro↑inv↑apo↓	(34)
Weishuang Xue	2018	Glioma	miR-190a-5p	-	Pro↑	(35)
			miR-488-3p			
Hongliang Li	2018	Lung cancer	miR-126	SLC7A5	Pro↑	(36)
Fanfei Kong	2018	Endometrial carcinoma	miR-195-5p	FGFR1, FGF2	Pro↑apo↓inv↑mig↑	(37)
Fengting Huang	2018	Pancreatic ductal adenocarcinoma	miR-20a-5p	ULK1	Autophagy↑pro↑	(38)
Yawen Ma	2017	Glioma	miR-186	Atg7, Beclin1	Angiogenic↑	(39)
Pindong Li	2017	Esophageal squamous	miR-203	LASP1	Pro↑	(40)
		Cell carcinoma				
S Zhang	2016	Cervical cancer	miR-200b	EZH2	Pro↑mig↑	(41)
Ying Chen	2018	Ovarian cancer	miR-214	EZH2	Pro↑inv↑mig↑	(42)
Xun Guo	2017	Hepatocellular carcinoma	miR-214	EZH2	Pro↑inv↑	(43)
Li Wan	2016	Non-small cell lung cancer	–	LATS2, P53	Pro↑	(44)
Weicong Li	2018	Clear cell renal cell carcinoma	–	BCL-2	Apo↓	(45)
Jing Zhao	2018	Gastric cancer	–	STAT3, VEGFA	Angiogenic↑	(46)
Midie Xu	2017	Gastric cancer	–	FOXM1	Pro↑inv↑	(47)
Di Cui	2016	Non-small cell lung cancer	–	P15, P21	Pro↑	(48)
Qinyi Zhou	2016	Thyroid cancer	–	EZH2, TSHR	Pro↑	(49)
Zhongwen Chang	2018	Prostate cancer	microRNA-186	EMT, Twist1	Inv↑mig↑	(50)
Xiangxiang Zheng	2016	Esophageal cancer	–	EMT	Inv↑	(51)
Yan Wang	2017	Breast cancer	–	SOX2	Pro↑inv↑	(52)
Meng Cui	2018	Nasopharyngeal cancer	miR-1207	–	Stem cell traits↑	(53)
Changyun Yu	2018	Squamous cell carcinoma of		β-catenin, EMT	Pro↑inv↑	(54)
		The head and neck				
Xingxing Zhang	2018	Pancreatic cancer	–	TGF β, Smad	Inv↑mig↑	(55)
Tonghai Huang	2017	Lung cancer	–	YY1	Pro↑inv↑	(56)
Enying Liu	2015	Ovarian cancer	–	p53, TIMP1	Drug resistance↑	(57)
Kazuhiro Yoshida	2017	Pancreatic cancer	–	EZH2, PRC2	Drug resistance↑	(58)
Guanfang Ping	2018	Colorectal cancer	–	-	Cisplatin resistance↑	(59)
Xianwen Zhang	2015	Gastric cancer	–	MDR1, MRP	Multidrug resistance↑	(60)
				mTOR, HIF-1α		
Heng Fan	2018	Colorectal cancer	–	–	Multidrug resistance↑	(61)

*Pro, proliferation; inv, invasion; apo, apoptosis; mig, migration. Increasing studies has demonstrated the oncogenic role of PVT1. PVT1 can interact with miRNAs and regulate gene transcription and protein expression, thereby exerting effects on cancer cells. The associated information was summarized of recent studies on PTV1 in cancer, including the first author, published year, the type of cancer, PVT1-binded miRNAs, PVT1-regulated protein and the functional role of these interactions*.

### *Myc-PVT1* Gene Pair

Amplification of the human 8q24.21 region has been shown to be common in various human cancers and to be associated with poor outcomes in cancer patients (62). Among the oncogenes in the 8q24.21 region, *Myc* is the best characterized, and it has a close relationship with the long non-coding RNA gene *PVT1* in terms of location and function. A synergistic effect in cancer initiation has been observed between Myc and PVT1 (63). Further analysis has identified RSPO1, a major regulator of the β-catenin signaling pathway, as a potential contributor to the regulation of Myc-PVT1 in tumor growth (17). Ramírez-Solis et al. have developed a mouse model with gain of Myc/PVT1/Ccdc26/Gsdmc, which shows increased oncogenic activity. Knocking down either Myc or PVT1 decreases proliferation of Myc/PVT1/Ccdc26/Gsdmc mutant tumor cells to the same extent as knocking down both PVT1 and Myc, thus suggesting the presence of a common oncogenic pathway shared by Myc and PVT1 in tumor initiation (64). PVT1/Myc co-operation plays a fundamental role in cancer associated with 8q24 gain. The dependence of the cancer driving effect of Myc on PVT1 has been observed in the HCT116 cell line (low copy number gain of 8q24), which overexpresses β-catenin, which in turn upregulates the downstream gene-*Myc*. CRISPR/Cas9-mediated PVT1 knockout of PVT1 in the HCT116 cell line blunts their tumorigenic ability and causes a 50% reduction in Myc protein levels (8, 63). Several other studies have indicated the complicated regulatory relationship between PVT1 and Myc in tumor initiation. SiRNA-mediated knockdown of PVT1 significantly decreases Myc protein. The results of chase experiments using cycloheximide suggest that PVT1 may help to maintain Myc protein stability through post-translational modification. Further analysis confirmed that PVT1 directly binds to Myc and consequently decreases phosphorylation of Myc at the threonine 58 residue, thereby inhibiting Myc degradation (65). In contrast to the observation in which PVT1 knockout induced a 50% reduction in Myc protein levels, siRNA-PVT1 has been found to significantly decrease Myc. This difference may be attributed to a compensation mechanism that may contribute to maintenance of Myc expression in a PVT1-independent manner, such as activation of the Wnt/β-catenin pathway. The significant decrease in Myc protein levels after siRNA-PVT1 treatment might be associated with the time point at which measurements are made. si-PVT1 may well lead to a significant decrease in Myc protein, but the compensatory mechanism may not completely reverse the effect. Interestingly, a recent study has confirmed the tumor-inhibitory role of the PVT1 promoter; the underlying mechanism is that the PVT1 promoter inhibits Myc transcription from the same chromosome through promoter competition (66). CRISPR-mediated inhibition of the 5′ transcription start site 1 (TSS 1) or transcription start site 2 (TSS 2) increases cancer cell proliferation, which is accompanied by increased expression of Myc. This phenomenon is attributable to the loss of interaction between the PVT1 promoter and PVT1 intragenic enhancer, which further facilitate the boundary of the Myc promoter and enhancer and increase Myc expression. In contrast to previous studies suggesting an oncogenic role of PVT1 and Myc in tumor progression, the work conducted by Cho et al. provided new insight into the functional role of the promoter of PVT1 in tumor initiation. Cells with PVT1 promoter mutation have better growth ability than cells with the wild type sequence. There is substantial interest in whether this tumor suppressor-PVT1 promoter might serve as a therapeutic target in PVT1 promoter-mutated cancers with Myc being the cancer driver. According to current understanding, the role of the PVT1-Myc pair is mainly based on the promotion of cancer cell proliferation. Close attention should be paid to the effects of proliferation in future research on PVT1 in cancers. Above all, the interaction between PVT1 and Myc plays a significant role in cancer initiation and progression, and disruption of this interaction seems to be a potential therapeutic target in cancer treatment.

### Modulation of miRNAs

#### Encoding miRNAs

Nearly all lncRNA are not translated into proteins, but the ability of miRNAs encoded by lncRNA genes to be translated has rarely been reported. Studies have shown that there are six miRNA sequences in the *PVT1* region in humans: miR-1207-5p, miR-1208, miR-1204, miR-1207-3p, miR-1205, and miR-1206 (67). MiR-1207-5p (68), miR-1204 (69), and miR-1205 (70) have been reported to exert oncogenic effects. In addition, a tumor-suppressive role has been found for miR-1207-3p (71) and miR-1205 (72). The six miRNAs are associated with the onset and progression of cancers. For instance, upregulated miR-1207-3p in prostate cancer cells can significantly inhibit migration, proliferation and promote apoptosis by targeting FNDC1 (71). Though a positive correlation of the expression levels of PVT1 and miR-1207-5p has been observed, the underlying mechanism remains unclear (18). Research has not only broadened understanding of lncRNA regulatory mechanisms in cancer but also clarified some previously unexplainable phenomena, such as the positive correlation between some lncRNAs and miRNAs. There must be some association between miRNAs and the oncogenic role of PVT1, which may constitute a novel oncogenic pathway. However, further exploration is necessary to provide a better understanding.

#### Acting as Endogenous Competitive RNAs (ceRNAs) or miRNA Sponges

CeRNA is a type of RNA transcript that binds to miRNA response elements (MREs) and decreases the levels of target RNAs, such as miRNA, mRNA and other RNAs with MREs (73). The ceRNA hypothesis was first presented to explain the relationship among various RNAs. Salmena et al. have hypothesized that all types of RNAs can communicate with one another by binding to MREs in 3′-UTRs, and this proposal has been verified experimentally (74, 75). CeRNA mechanism is also reported to be utilized by PVT1 to regulate gene expression. PVT1 can relieve the miRNA-related suppressive effect on target genes by sponging corresponding miRNAs.

The biological effects of PVT1 in cancer may occur through sponging miRNA directly. For instance, several studies have demonstrated that miR-195 plays a crucial role in cancer initiation and development (76, 77). MiR-195 was one of the earliest miRNA targets identified for PVT1, and Wu et al. have reported that PVT1 decreases radio-sensitivity in non-small cell lung cancer (NSCLC) by sponging miR-195 (19). Cell cycle arrest and apoptosis can be induced by either silencing PVT1 or overexpressing miR-195 in osteosarcoma (20). In cervical cancer and glioma, PVT1 improves tumor growth by negatively regulating miR-424 (21, 22). Similar mechanisms have been reported for PVT1-miR-146a, PVT1-miR-497, PVT1-miR-26b, and PVT1-miR-133a (10, 23–26). Among the affected miRNAs, miR-26b and miR-195 have received substantial attention, and lncRNA/miRNA cooperation is now well-known to have promising prospects in cancer research.

#### Regulation of Protein Levels Through a miRNA Approach

In either physiological or pathological conditions, cellular biological behaviors cannot continue without various proteins. PVT1 regulates the expression of some vital proteins by downregulating miRNAs. Among these proteins, Hypoxia-inducible factor-1 (HIF-1α), and Runt-related transcription factor 2 (RUNX) are two common proteins.

HIF-1α is an important factor that contributes to maintaining cell homeostasis in low-oxygen environments. Whereas, hypoxia can induce expression of many oncogenes and suppress tumor suppressor genes, the overexpression of HIF-1α contributes to the initiation and progression of various cancers. PVT1 upregulates the expression of HIF-1α by binding to miR-199-5p and miR-186, thus further facilitating the proliferation of cancer cells (27, 28). In addition, PVT1 promotes colon cancer cell proliferation through releasing the inhibitory effect of miR-30d-5p on RUNX2, a crucial transcription factor associated with tumor cell growth, proliferation and metastasis (29). Moreover, PVT1 accelerates cell proliferation, migration and invasion of colorectal cancer cells *in vitro*, and tumorigenesis *in vivo*, with the underlying mechanism of simulating RUNX2 expression by inhibiting miR-455 activity. Interestingly, upregulation of RUNX2 can lead to increased expression of PVT1, thus suggesting a positive feedback loop between PVT1 and RUNX2 (30). Moreover, PVT1 upregulates the expression of matrix metalloproteinase (MMP)-9, thus increasing invasion ability in NSCLC by inhibiting miR-200a and miR-200b (31). Similar mechanisms have also been found in PVT1-miR-448-SERBP1 (32), PVT1-miR-34-CDK1 (33), PVT1-miR-497-HK2 (34), and others (20, 35–40, 78).

### Regulation of Gene Transcription and Protein Levels

Although the cytoplasmic level of PVT1 holds a dominant position, and PVT1 exerts regulatory effects mainly through modulation of miRNAs, PVT1 also can regulate gene transcription and protein levels in the cell nucleus and extracellular fluid.

#### Gene Transcription

EZH2 is a multifunctional molecule that has an oncogenic function in various cancers (79). As a key element of Polycomb repressive complex 2, EZH2 plays an important role in catalyzing trimethylation of histone H3 lysine 27 (H3K27me3). PVT1 regulates gene transcription through EZH2. For instance, PVT1 forms a complex with EZH2 and then recruits EZH2 to the promoter regions of miR-195 and miR-200b, increases the trimethylation of histone H3K27, and downregulates the levels of miR-195 and miR-200b in cervical cancer (9, 41). Additionally, PVT1 inhibits miR-214 expression by interacting with EZH2 in ovarian cancer and hepatocellular carcinoma (42, 43). In this manner, PVT1 influences the levels of downstream miRNAs, thus regulating the biological behavior of cancer cells. MiR-200b is associated with cervical cancer cell proliferation, invasion and migration, whereas miR-195 is associated with epithelial-mesenchymal transition (EMT) and chemoresistance. By reducing the level of miR-200b and miR-195, PVT1 promotes progression and chemoresistance in cervical cancer.

Beyond regulating gene transcription of miRNAs, PVT1 recruits EZH2 to mRNA promoters, thus inhibiting transcription. For instance, PVT1 inhibits the expression of large tumor suppressor kinase 2 (LATS2) in NSCLC by recruiting EZH2 to the LATS2 promoter (44). This evidence together suggests a high potential for clinical application, through targeting the PVT1/EZH2 axis to regulate gene transcription (80), and further study may be needed to analysis the binding sites of PVT1 inside EZH2, facilitating the development anti-cancer therapies targeting the PVT1/EZH2 axis.

#### Protein Levels

Beyond miRNA regulation, PVT1 can bind proteins and influence protein levels directly. For instance, epidermal growth factor receptor (EGFR) is a downstream target of PVT1 that normally regulates cell apoptosis and proliferation. PVT1 inhibits apoptosis and cell cycle arrest in clear cell renal cell carcinoma through increasing the expression of EGFR (45). Zhao et al. have reported the regulatory role of PVT1 in tumor angiogenesis; the underlying mechanism is that PVT1 interacts with p-STAT3 protein, thereby maintaining its stability, and further enhances VEGFA promoter activity to stimulate VEGFA expression (46). Forkhead box M1 (FOXM1) is a member of the Forkhead box transcription factor family, which plays critical roles in tumor initiation, proliferation, metastasis and drug resistance (81). PVT1 directly binds to FOXM1 and enhances its stability, and FOXM1 in turn initiates PVT1 expression through binding to the PVT1 promoter, thus forming a feedback loop between PVT1 and FOXM1 that plays a critical role in promoting gastric cancer cell proliferation and invasion (47). In NSCLC, PVT1 also promotes cancer cell proliferation and cell cycle progression through inhibiting expression of p15 and p21, two growth inhibitors of cell cycle checkpoints (48).

The cooperation of thyroid-stimulating hormone (TSH) and thyroid-stimulating hormone receptor (TSHR) is well-known to play an important role in the regulation of thyroid cancer cell proliferation. Zhou et al. have reported that PVT1 increases the expression of TSHR, thereby exerting an oncogenic function in thyroid cancer (49). However, the specific regulatory mechanism is unclear and need to be explored. There are various receptors on the cell surface that remain signal transmission and support cellular biology. Over-activating or silencing these receptors may facilitate a variety of pathological processes, including cancer initiation, and progression (82). Although this mechanism has rarely been reported for PVT1 to date, it may be a novel research direction.

### Induction of EMT

EMT plays a vital role in cancer cell metastasis and invasion, and upregulation of EMT has been detected in almost all types of cancer (83). E-cadherin and vimentin are two critical proteins that are universally considered EMT markers. Vimentin induces, whereas E-cadherin inhibits, EMT. Many lncRNAs, including PVT1, are related to EMT through these two proteins. PVT1 has been reported to sponge miR-186; to downregulate E-cadherin but upregulate vimentin, snail and Twist1; and to induce EMT and promote the progression of prostate cancer (50). Zheng et al. have reported that PVT1 increases esophageal cancer invasion ability by upregulating the EMT, but specific targets have not been found (51). The same situation has also been found in breast cancer (52).

### Regulation of Signaling Pathways

Various signaling pathways existing in cells play a vital role in the process of tumorigenesis (84). Among the PVT1-affected proteins regulated either directly or through miRNA pathways, some may be involved in signaling pathways. The associated signaling pathways have been found to include the phosphatidylinositol 3-kinase (PI3K)/AKt cascade (53), mitogen-activated protein kinase/extracellular signal-regulated (MAPK/ERK) pathway (37), Wnt/β-catenin pathway (54), P53 pathway (44), and TGF β/Smad pathway (55).

#### PI3K/AKt Cascade and MAPK/ERK Pathway

The significant activation of the PI3K/AKt cascade has been detected in the progression of various cancers. During the stimulation process, PI3K activates AKt protein, and the activated AKt then transfers to the nucleus, where it regulates cellular proliferation, invasion, metabolic reprogramming, migration, autophagy and caducity, and exerts oncogenic effects (85). The MAPK/ERK pathway is tightly associated with the PI3K/AKt cascade, and it normally is thought to exert important effects in the development of cancer. Many substrates of ERK phosphorylation have been found to contribute to cell proliferation and invasion after activation of the signaling pathway (86). Fibroblast growth factor receptor (FGFR1) and basic fibroblast growth factor (FGF2), activating factors of PI3K/AKT and the MAPK/Erk cascade, are targets of miR-195-5p. PVT1 downregulates miR-195-5p by sponging it, increases the expression of FGFR1 and FGF2 and subsequently activates the PI3K/AKT and MAPK/Erk pathways in endometrial carcinoma (37). In nasopharyngeal cancer, PVT1 promotes cancer stem cell-like traits by downregulating miR-1207 and further induces the activation of the PI3K/AKt cascade (53).

#### Wnt/β-Catenin Signaling Pathway

As one of the most important signaling pathways, the Wnt/β-catenin pathway exerts vital effects in mediating oncogenesis and development. Moreover, increasing evidence indicates that the Wnt/β-catenin pathway members have high potential as therapeutic targets for cancer treatment. In squamous cell carcinoma of the head and neck, PVT1 can promote EMT and increase cancer stem cell-like properties by activating the Wnt/β-catenin pathway (54).

#### P53 Pathway

The transcription factor p53 is an important tumor suppressor, and overexpression of p53 significantly contributes to cancer cell apoptosis, promotes cell cycle arrest and inhibits cancer progression (87). In NSCLC, PVT1 downregulates the expression of LATS2 and further decreases p53 levels, inhibits apoptosis and promotes cell proliferation (44). Although these findings suggest a novel signaling pathway associated with PVT1, the direct relationship between PVT1 and p53 requires further research.

#### TGF β/Smad Pathway

The TGF β/Smad cascade, one of the classical signaling cascades, is a tumor suppressor pathway closely associated with organ fibrosis and oncogenesis. In pancreatic cancer, PVT1 increases p-Smad2/3 and TGF-β1 expression, promotes EMT and increases invasion and migration abilities (55).

## Clinical Applications in Cancer

Because lncRNA PVT1 plays a vital role in cancer, studies on the underlying mechanisms suggest promising clinical applications. According to current research, there are three main applications: diagnostic biomarkers, prognostic biomarkers and therapeutic targets. Among them, drug resistance related applications have received the most attention and development.

### Diagnostic Biomarkers

Although great progress has been made in cancer therapy, the overall survival rate, and quality of life for cancer patients remains a major challenge. The poor prognosis of cancer patients may be mainly attributed to drug resistance, metastasis and frequent tumor relapse, which are closely associated with advanced diagnosis. Thus, there is an urgent need to increase the early stage diagnosis rate to enable timely treatment for cancer patients and improve their prognosis. In the past few years, scientists have tried a variety of methods, such as radiological technology, immunology and biomarkers, to improve diagnosis and monitoring of early stage cancer. Among them, diagnostic biomarkers have received extensive attention, and various studies have shown that many lncRNAs have great potential as diagnostic biomarkers, including PVT1 (88).

Because of the upregulated expression of PVT1 in cancers, many studies have predicted and validated its potential for clinical application as diagnostic biomarkers (16, 55). Moreover, as one of the most established lncRNAs, PVT1 exhibits better diagnostic power than other lncRNAs. A meta-analysis has shown that PVT1 has moderate value in discriminating HCC from control with a summary receiver operating characteristic of 0.81 (95%CI: 0.77–0.84), thus suggesting the potential of PVT1 as a diagnostic indicator for liver cancer (89). In melanoma, the area under the receiver operating characteristic curve value of serum PVT1 has reached 0.937, and PVT1 shows a good correlation with cancer cell proliferation and metastasis (11). Furthermore, the cooperation between PVT1 and traditional diagnostic biomarkers, such as carcinoembryonic antigen (CEA) and carbohydrate antigen 19-9 (CA19-9) may improve diagnostic power. There are several advantages of utilizing PVT1 as a diagnostic biomarker of cancer. First, PVT1 plays an important regulatory role in the initiation and development of cancer; second, PVT1 can be detected in cancer tissue; third, PVT1 possesses cancer specificity; fourth, PVT1 is a novel cancer diagnostic biomarker with high specificity and easy detection in the serum, plasma and saliva. In addition, the inspection of PVT1 in blood or other body fluid is non-invasive. A variety of studies have shown that using serum lncRNA as a diagnostic biomarker is feasible for cancer diagnosis (90). Although great progress has been made, further studies related to clinical applications are needed.

### Prognostic Biomarkers

With the rapidly growing incidence and mortality of cancer, its overall prognosis will be a leading determinant of public health and life expectancy worldwide. A surgical operation is the most effective method for cancer treatment, but relapse and metastasis severely affect the prognosis (1). In recent years, some studies have shown that PVT1 is closely associated with clinicopathological features and can act as a prognostic biomarker in cancer. There is no doubt that obtaining more prognostic information from PVT1 would contribute to the treatment of cancer.

Using TCGA cohorts, Posa et al. found that more than half of the patients presented Myc-PVT1 locus amplification, and PVT1 upregulation has been observed in almost all tumors when compared with surrounding normal tissues. Kaplan-Meier survival analysis has shown a negative correlation between PVT1 expression and the survival rate of patients, thus indicating the potential for its application as a prognostic biomarker (91). Li et al. have reported that detection of PVT1 expression in the serum might be feasible in diagnosis of breast cancer. Moreover, PVT1 expression in plasma has a positive correlation with histological grade, expression of Ki-67, tumor size and lymph node metastasis, and might act as an independent prognostic factor for the survival of breast cancer patients (92).

### Therapeutic Targets

With the improved understanding of the pathogenesis of cancer, many molecules, and signaling pathways may be suitable for targeted therapies. Because PVT1 exerts oncogenic effects related to many molecules and cascades, it has been regarded as an ideal therapeutic target and this has been validated in recent studies. Targeted PVT1 knockout can be utilized to improve radiotherapeutic sensitivity (19), inhibit cancer metastasis (31), block the growth of cancer *in vivo* (56), promote cancer cell apoptosis (18), and reverse drug resistance (57). Among all the applications, drug resistance related applications are the ones which should best be developed. Substantial studies have shown that PVT1 can reverse drug-resistance in cancers, and some chemotherapeutic drugs can exert anti-cancer effects through PVT1, such as gemcitabine (58), carboplatin and docetaxel (57), paclitaxel (9), and cisplatin (59). These drugs provide a possibility for chemotherapy related clinical applications, and studies have indicated that knocking out PVT1 can reverse multidrug resistance in gastric cancer and colorectal cancer (60, 61). Nonetheless, further information is needed to determine the therapeutic value of PVT1 for the treatment of cancers.

## Conclusions

We summarized multiple mechanisms related to lncRNA PVT1 in cancer cells as the basis of molecular targets and highlighted its potential clinical application value. Studies have found that PVT1 influences proliferation, invasion, metastasis, drug resistance and angiogenesis in various cancers via the Myc-PVT1 pair, the modulation of miRNAs, and the regulation of gene transcription and protein levels. Regarding clinical applications, PVT1 shows good prospects for drug resistance related applications. Our review should aid in the understanding of PVT1 and PVT1-based therapy in cancer. Further studies, especially clinical studies, are needed to verify the clinical value of PVT1.

## Author Contributions

M-YL, X-HT, YF, T-JW, and J-MZ substantially contributed to the conception, drafting, editing, and final approval of this manuscript.

### Conflict of Interest Statement

The authors declare that the research was conducted in the absence of any commercial or financial relationships that could be construed as a potential conflict of interest.
